# Quadriceps Tendon Delamination Tear: A Case Report

**DOI:** 10.7759/cureus.48061

**Published:** 2023-10-31

**Authors:** Alexandros Tsioupros, Constantinos Chaniotakis, Vassileios Genetzakis, Chrysostomos Tsatsoulas, Ioannis M Stavrakakis

**Affiliations:** 1 Orthopedics and Traumatology, Venizeleio General Hospital, Heraklion, GRC

**Keywords:** quadriceps tendon, extensor mechanism knee injuries, insall-salvati ratio, tendon tear, delamination

## Abstract

Partial quadriceps tendon ruptures are rare and they are usually managed non-operatively, provided that the extensor mechanism is intact. In case the extension mechanism is compromised, a more aggressive treatment is required, which includes surgical repair of the tendon.

We present an extremely rare case of a 42-year-old male lifter who sustained a quadriceps tendon delamination tear, after lifting weights. Careful clinical examination revealed a compromised extension mechanism of the knee. Proper imaging confirmed the diagnosis of partial but significant rupture of the undersurface of the quadriceps tendon, which was treated operatively (standard tendon repair with the Krakow technique and three transosseous tunnels) with a very good outcome.

## Introduction

Quadriceps tendon rupture is considered a rare injury, with an incidence of 1.37/100000 patients per year. It most commonly affects middle-aged males with mean age being 51.1 years, peaking in the seventh decade [1]. There are various mechanisms reported with the most common being simple fall (61.5%) followed by fall from stairs (23.4%), rupture during sports (6%), and other less common causes like spontaneous ruptures, car accidents, agricultural injuries, and non-penetrating blow [1]. Indirect violent eccentric contraction of the quadriceps is the usual cause of rupture [2]. Many factors have been associated with an increased risk of quadriceps tendon ruptures, such as age >40, steroid injections, previous patellar tendinopathy or repetitive micro-trauma, diabetes, obesity, inflammatory arthritis, hyperparathyroidism, chronic renal failure, and fluoroquinolones (especially levofloxacin) [3].

The quadriceps tendon is formed by three separate tendinous laminae [4]. Complete quadriceps tendon ruptures are treated operatively and require intervention ideally within two weeks of injury [5]. Partial ruptures can be treated conservatively depending on the extent of rupture as well as the resulting functional disability. Conservative treatment includes cast immobilization with the knee in full extension for six weeks, followed by a removable splint [2,6].

## Case presentation

A 42-year-old male powerlifter presented to the emergency department (ED) in a wheelchair after he had lifted 110 kg using the “clean and jerk” technique at the gym. He mentioned a right-sided, acute, above-the-knee pain while lifting the weights. No comorbidities were noted in his past medical history and he denied any anabolic steroid or other medication use. In addition to this, the patient did not mention any history of knee pain or trauma before the injury.

**Figure 1:**
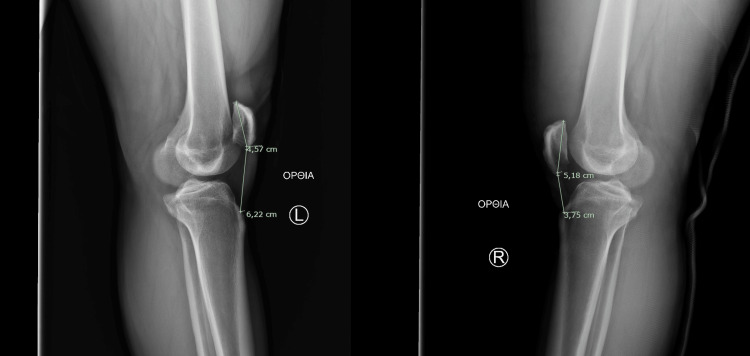
Knee X-ray with Insall-Salvati ratio Comparison between right knee (affected) and left knee (unaffected).

On physical examination, severe swelling of the knee joint was noted. No palpable gap could be felt at the superior pole of the patella. An extension lag of 30° was identified, indicating a compromised knee extension mechanism. Plain radiographs were performed at the ED, which revealed patella baja of the right knee. Insall-Salvati ratio was calculated, confirming the patella baja of the right knee with an Insall-Salvati ratio of 0.72 compared to 1.36 of the unaffected left knee (Figure 1). No avulsion fractures were present. 

**Figure 2:**
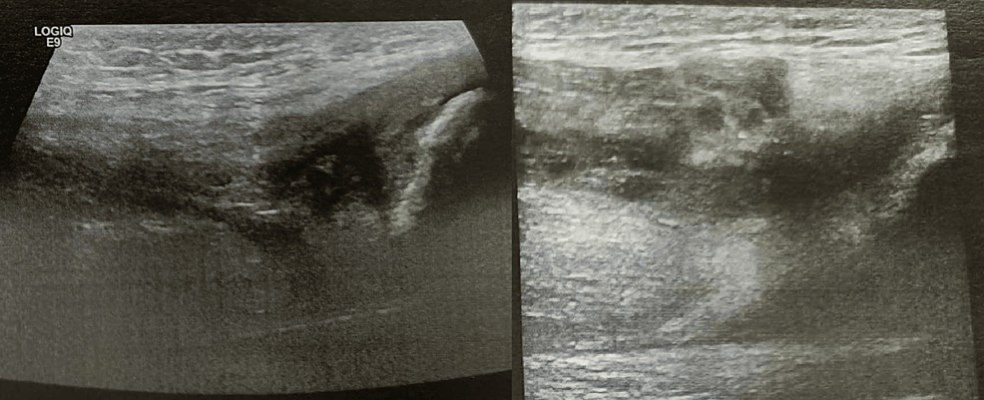
Ultrasound scan

**Figure 3:**
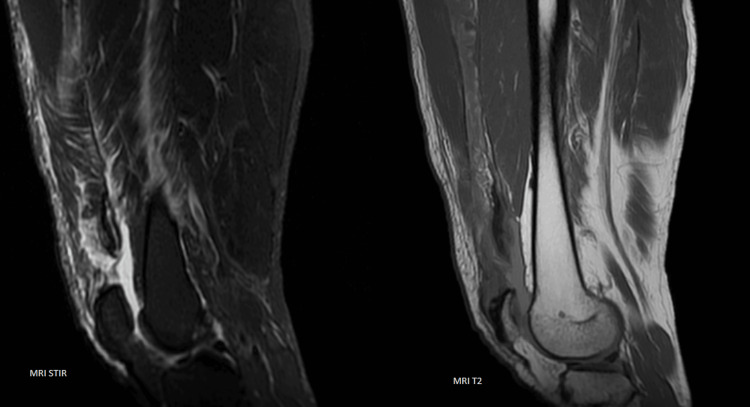
MRI scan (STIR and T2 views) MRI, magnetic resonance imaging

In the acute setting, the knee was immobilized using a backslab in full extension allowing the patient to bear weight on the injured limb. Further assessment included an ultrasound scan (Figure 2) and magnetic resonance imaging (MRI) (Figure 3), which revealed a complete tear of the vastus lateralis, vastus medialis, and vastus intermedius tendons, whereas the rectus femoris tendon remained intact.

**Figure 4:**
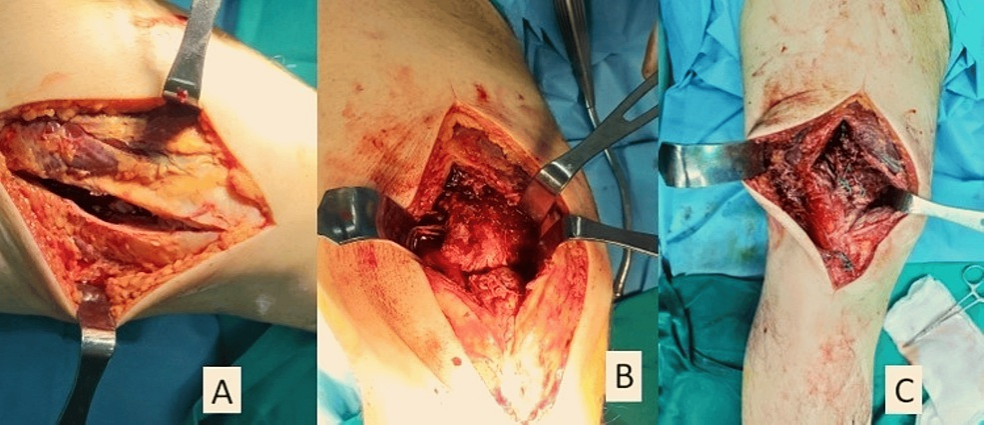
Surgical procedure Longitudinal incision of the rectus femoris tendon (A). Complete rupture of the deep quadriceps tendon layers (B). Surgical repair (C)

Due to the significant rupture of the tendon, operative treatment was obtained. The surgical technique chosen included the use of transosseous tunnels. A midline longitudinal approach was performed, giving access to the injured quadriceps tendon and the inferior pole of the patella. As expected from the ultrasound scan, the superficial layer of the quadriceps tendon was found to be intact. A longitudinal incision of the rectus femoris tendon was performed (Figure 4A) and it revealed complete rupture of the deep quadriceps tendon layers (Figure 4B). Debridement of the tendon edges as well as the superior pole of the patella was performed followed by the application of two No.5 ethibond sutures in the medial and the lateral half of the tendon using the Krakow technique. Three transosseous tunnels were created, through the length of the patella in which the sutures passed and then tied distally, securing the tendon to its attachment site (Figure 4C). 

He was then placed in a hinged knee brace locked in full extension followed by gradual unlocking and was allowed full weight bearing on the affected limb. The knee was locked in full extension for two weeks, followed by unlocking the brace, allowing 30° of flexion for another two weeks. Finally, 60° of flexion was allowed for the last two weeks. The brace was then removed, and physiotherapy was initiated.

Six months after the surgery the patient had established a full range of motion of the knee and a quadricep muscle strength of 5/5 according to the Oxford muscle scale. At this point, he had returned to work, and he was able to perform recreational activities. On his final follow-up one year postoperatively, the patient was able to perform a full weight training program at the gym. The International Knee Documentation Committee (IKDC) score was calculated and was found to be 81.6 and 92.0 at six months and at one year postoperation, respectively.

## Discussion

Partial quadriceps tendon tears are rare injuries, which can lead to significant disability if not treated properly. Management can be either conservative or operative depending on the amount of rupture and its clinical impact. Partial quadriceps tendon tear with delamination of the undersurface is even rarer. Thorough clinical examination and imaging are of paramount importance in order not to miss such injuries. Hematoma formation and swelling may hinder the tendon defect on examination. In addition, partial tears may preserve some degree of function, only lacking a few degrees of extension. Early diagnosis and treatment are crucial, as quadriceps muscle retraction and subsequent atrophy can be established rapidly, affecting the functional outcome significantly [2].

In our case, the diagnosis could be easily missed, due to the absence of a palpable gap and the presence of a nearly complete active knee extension. Appropriate assessment and management of the patient led to a satisfactory clinical outcome. The surgical technique including the use of transosseous tunnels was chosen since it is considered to have fewer complications than the suture anchor technique [7].

To the best of our knowledge, this is the second case of quadriceps tendon delamination described in the literature [8]. Our case of delamination had an extension lag of 30°, in contrast to the case already published that had suffered complete extensor mechanism failure. Loose et al. in a retrospective analysis reported a mean IKDC score of 73.1 (38.0-87.0) from patients being a minimum of 40 years old who received quadriceps tendon refixation following acute quadriceps tendon rupture (mean follow-up: 7.2 years) [9]. Chang et al. in a retrospective review compared five patients with bilateral quadriceps tendon ruptures to an age-matched control group of five patients with similar medical history, who sustained unilateral quadriceps tendon ruptures. All patients underwent surgical repair with tranosseous tunnels. A mean IKDC (mean follow-up: 25.4 months) of 71.9 (range: 34.4-91.6) was reported in patients with bilateral quadriceps tendon repairs, while the age-matched control group had a mean IKDC score of 88.3 [10].

## Conclusions

Quadriceps tendon delamination tear is very rare, and it can be easily missed. Our case highlights that proper examination and imaging are of paramount importance in order to provide the appropriate treatment and achieve a good outcome. Our case report is the second case described in the literature, and we believe that this topic should be further researched.
